# Biomechanical Characterisation of the Human Auricular Cartilages; Implications for Tissue Engineering

**DOI:** 10.1007/s10439-016-1688-1

**Published:** 2016-07-14

**Authors:** M. F. Griffin, Y. Premakumar, A. M. Seifalian, M. Szarko, P. E. M. Butler

**Affiliations:** 1 UCL Division of Surgery & Interventional Science, Centre for Nanotechnology & Regenerative Medicine, University College London, London, UK; 2 Anatomical Sciences, Institute for Medical and Biomedical Education, St. George’s, University of London, London, UK; 3 Department of Plastic and Reconstructive Surgery, Royal Free Hampstead NHS Trust Hospital, London, UK

**Keywords:** Elastic Modulus, Stress–strain, Human cartilage, Auricular, Chondrocyte

## Abstract

Currently, autologous cartilage provides the gold standard for auricular reconstruction. However, synthetic biomaterials offer a number of advantages for ear reconstruction including decreased donor site morbidity and earlier surgery. Critical to implant success is the material’s mechanical properties as this affects biocompatibility and extrusion. The aim of this study was to determine the biomechanical properties of human auricular cartilage. Auricular cartilage from fifteen cadavers was indented with displacement of 1 mm/s and load of 300 g to obtain a Young’s modulus in compression. Histological analysis of the auricle was conducted according to glycoprotein, collagen, and elastin content. The compression modulus was calculated for each part of the auricle with the tragus at 1.67 ± 0.61 MPa, antitragus 1.79 ± 0.56 MPa, concha 2.08 ± 0.70 MPa, antihelix 1.71 ± 0.63 MPa, and helix 1.41 ± 0.67 MPa. The concha showed to have a significantly greater Young’s Elastic Modulus than the helix in compression (*p* < 0.05). The histological analysis demonstrated that the auricle has a homogenous structure in terms of chondrocyte morphology, extracellular matrix and elastin content. This study provides new information on the compressive mechanical properties and histological analysis of the human auricular cartilage, allowing surgeons to have a better understanding of suitable replacements. This study has provided a reference, by which cartilage replacements should be developed for auricular reconstruction.

**Electronic supplementary material:**

The online version of this article (doi:10.1007/s10439-016-1688-1) contains supplementary material, which is available to authorized users.

## Introduction

Microtia, translated from the Greek, means ‘little ear’ and is the medical word used to describe a small or absent ear in newborn babies.^11^ Affecting one in 6000 live births, microtia can appear in isolation or as a feature of other syndromes such as Hemifacial microsomia or Treacher collins syndrome.^11^ Currently, the gold standard surgical technique for ear reconstruction is using autologous rib cartilage.^16^ The first stage involves carving and joining together the rib cartilage to create a framework to replicate a new ear.^16^ There are a number of complications with this reconstructive technique,^11^ including the surgery must be delayed until the child is 6–10 years,^16^ associated cartilage donor site risks including pneumothorax and chest wall deformities^7^ and rib cartilage can warp over time.^2^,^7^,^18^,^23^ In the motivation to avoid extracting costal cartilage and provide earlier surgical intervention, alloplastic materials are currently being used to reconstruct the ear. Synthetic biomaterials offer a number of advantages including the mass production of implants with various predetermined shapes and sizes enabling ‘off-the-shelf’ products and decreased donor site morbidity.^2^,^3^

When designing an auricular implant for clinical application, there are several key characteristics of the material that need to be considered.^3^ The surface and bulk properties of the synthetic material need to be suitable to allow for good tissue integration and angiogenesis. When considering the bulk properties of a material, one important characteristic is the mechanical properties of the material. It is known that to prevent mechanical mismatch, a synthetic material should have a similar Elastic Modulus to the tissue it is replacing.^8^,^14^,^21^ The mechanical properties of the human auricular cartilages has not been fully investigated to date which makes it difficult to produce an auricular construct with similar properties as the native tissue it is required to replace. We have already characterised the mechanical properties of the human nasal cartilages, developing a reliable method to characterise the mechanical properties of the human facial cartilages.^5^ The aim of this study was to create an (a) map of the mechanical properties of human auricular cartilage and to support these findings with a (b) biomechanical and histological map of extracellular matrix components.

## Materials and Methods

### Cartilage Harvest

Fresh-frozen human auricular cartilage was harvested from the ear structures of the 15 male cadaveric specimens (average 59 ± 10 years). Following harvest, the auricular construct was placed into sterile normal saline at 37.5 °C to defrost the auricular cartilages for further dissection. Firstly, the skin and fascia were dissected from the cartilaginous framework. Following this procedure, the cartilage specimens were dissected into 14 areas as described below for mechanical testing.

### Mechanical Testing

**Figure 1:**
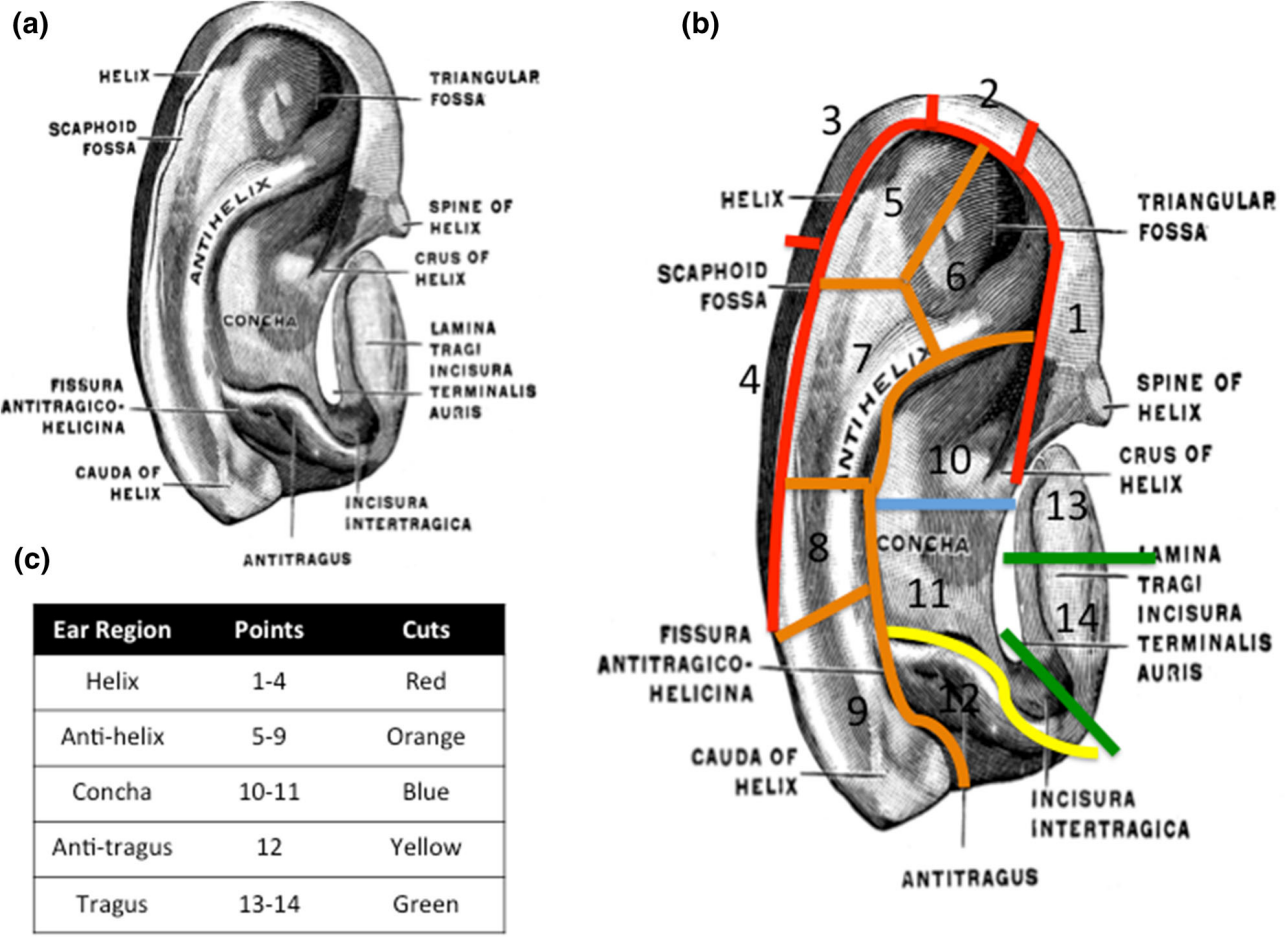
Protocol by which the auricular cartilages were dissected according to the 14-point map. (a) Ear anatomy prior to dissection. (b) Ear regions with their associated points. (c) Table demonstrating the points and regions of the ears tested. Taken with permission from Gray, Henry Gray’s Anatomy: Descriptive and Applied (Philadelphia: Lea & Febiger, 1913), p. 1120, http://etc.usf.edu/clipart/54100/54112/54112_ear.htm, accessed on 8 July 2016.

After removal of the skin and fascia, the auricular cartilages were tested as illustrated in Fig. 1. Once the ear was fully denuded the following guidelines were utilised referring to Fig. 1 to cut the ear into 14 designated areas. The 14 points were initially chosen to provide the most detailed mechanical and histological map of the auricle, which covered all the anatomical structures of the ear including the helix, antihelix, concha, anti-tragus and tragus. After observing no differences in the mechanical properties of the auricular cartilage, the data was reanalysed and a five-point map was further used including the helix, antihelix, concha, tragus and antitragus to reflect the anatomical structures of the auricle (Fig. 1). The thickness of the auricular cartilage was measured using digital Vernier callipers. All slices were similarly oriented so that the anterior surface would be tested under compression along the same directional axis. Cartilage samples were tested using indentation compression using a Mach-1 materials testing machine (Biomomentum, Canada). Each sample was loaded to 300 g at 1 mm/s *via* the 1 kg load cell. The semi spherical indenter was 0.2 mm in diameter. After the 300 g was reached, the tissue was allowed to relax for 15 min (a time point sufficient to control for stress equilibrium).

The indenter was chosen for all samples as cartilage sample that are approximately 8 times greater diameter than the indenter will react as if it were part of an indefinite sample and create appropriate boundary conditions.^17^ Using an indenter much smaller than the radius of the cartilage sample diameter eliminated any edge effects. The resulting Young’s Elastic Modulus and stress relaxation properties calculations were calculated as previously described 19 and shown in brief in Supplementary Fig. 1. In addition to Young’s Elastic Modulus, the stress-time slope was used to measure the rate of loading of the anatomical ultrastructure (i.e., removal of strain which normalizes thickness to displacement).

#### Cartilage Preparation

A. Helix cuts
  i. An incision was made from the lateral external ear along the superior border of the antihelix superior crus, into the curve of the helix. The helix was then divided into four by measuring the height and length using electronic calipers. The centre of the four points was points 1––4 as shown in Fig. 1.
B. Antihelix cuts
  i. Using the triangular fossa as the junction between the superior and inferior crus, the superior crus and inferior crus were dissected. The centre of each of these points was points 5 and 6.
  ii. Point 7 was identified by dissecting along the inferior border of the curve that is visible at the intercrus junction.
  iii. The remaining point of tissue was divided into half by length and width to create points 8 and 9.
C. Antitragus cuts
  i. A circumference around the prominent antitragus was cut allowing for a final tissue circumference, which met the required boundary conditions. The centre of this antitragus point was point 12.
D. Tragus cuts
  i. The tragus was first cut from the concha by making a longitudinal incision across the isthmus.
  ii. Measuring the width and height using electronic calipers cut the tragus into two areas. The centre of the two-tragus points was points 13 and 14.
E. Concha cuts
  i. Measuring the width and height using electronic calipers then cut the free concha. The centre of each of these concha points was points 10 and 11.

### Histological Testing

Tissue was formalin-fixed, paraffin-embedded and sectioned at 8 *μ*m. Histological analysis including Haematoxylin and Eosin (H&E) for general structure, Alician blue & Periodic Acid-Schiff (PAS) staining for glycosaminoglycans and proteoglycans and Elastin Van Gieson (EVG) staining for elastin and collagen type I. All stains were conducted according to standard protocols, then photographed with a slide scanner (Nanozoom Slide Scanner) at ×10 magnification.

### Data Analysis

The differences between the auricular cartilages in the Youngs Elastic Modulus, final stress relaxation rate and final absolute relaxation were analysed statistically using one-way analysis of variance (ANOVA) with Tukey HSD *post hoc* analysis (JMP, v10; North Carolina, USA). Significance was described as *p* < 0.05. Kaleida-graph (v.4.1, Pennsylvania, USA) was used for graphically representing data.

## Results

### Mechanical Testing

**Figure 2:**
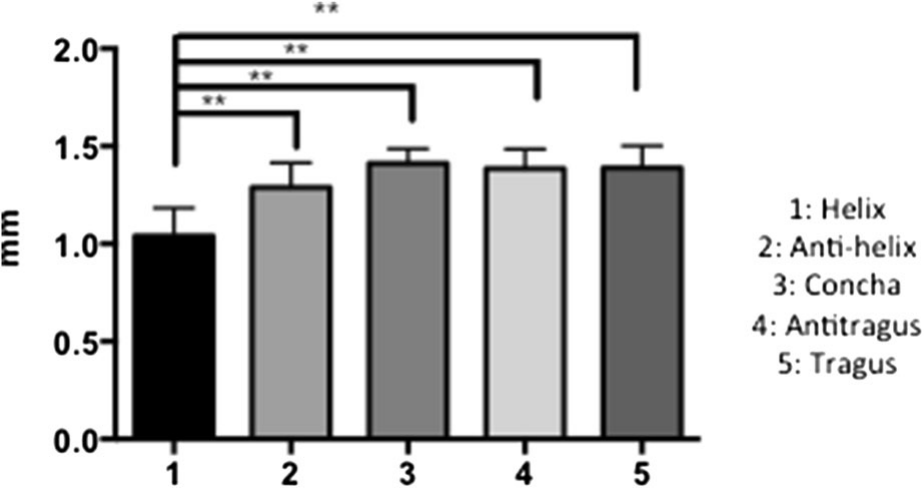
Thickness of the auricular cartilages groups after dissection (mm). *p < 0.05; **p < 0.01; ***p < 0.001.

The thickness of each of the auricular cartilages was carried out using electronic callipers (Fig. 2). The helix and antihelix were significantly thinner than the concha, antitragus and tragus cartilage (*p* < 0.01).

**Figure 3:**
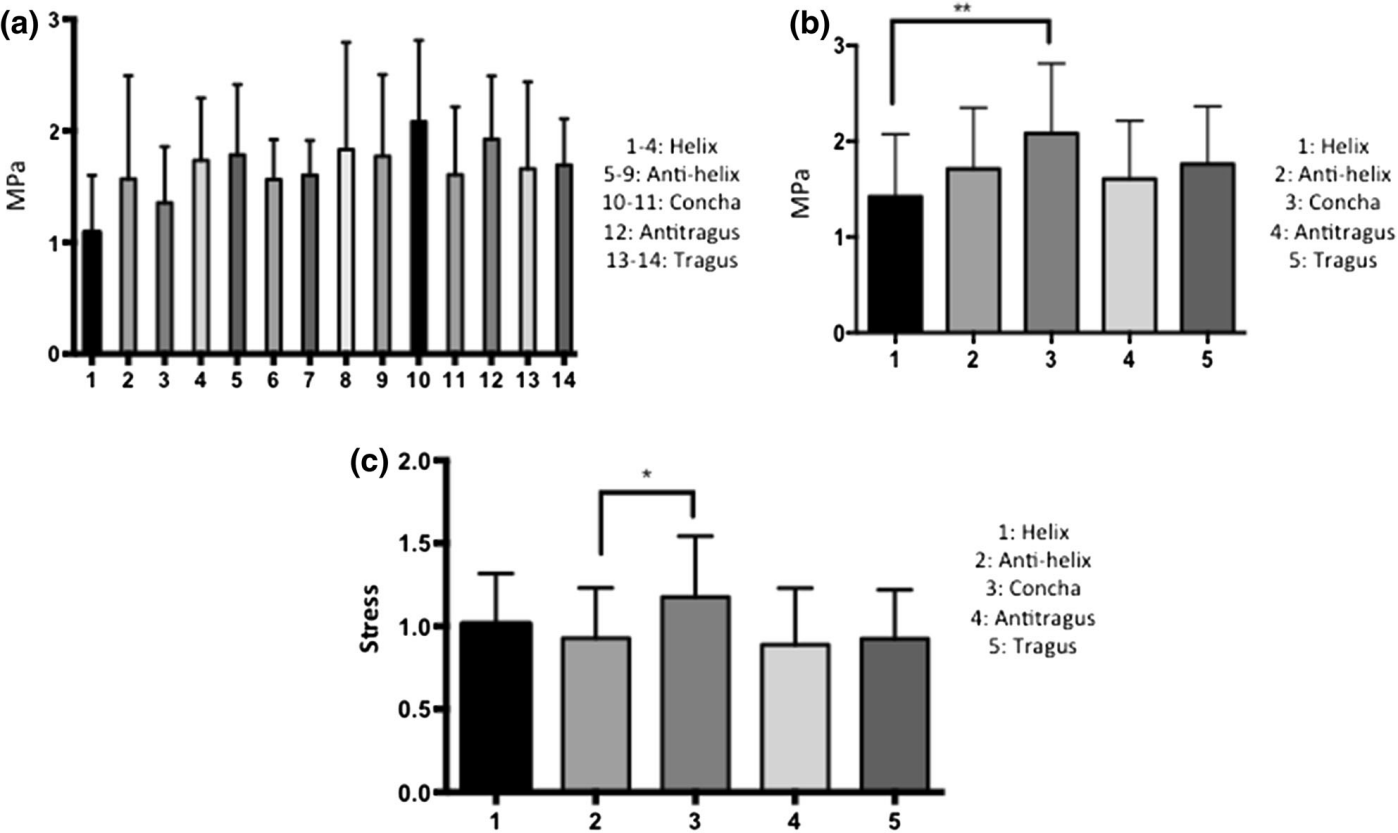
Compression Young’s Elastic Modulus of the auricular cartilages according to the (a) 14-point map and (b) 5-point map. (c) Rate of loading based on the anatomical structure of the auricular cartilages. *p < 0.05; **p < 0.01; ***p < 0.001.

A 14-point anatomical map was elucidated to provide a compressive mechanical map of the auricular cartilage. A compression Elastic Modulus was formulated for each of the auricular cartilage areas according to the 14-point anatomically based map (Fig. 3a). Using this 14-point map as a base for comparisons, there were no significant differences among the auricular cartilage regions with an average overall Young’s modulus of 1.66 ± 0.63 MPa (Fig. 3a). Observing no difference amongst the 14 cartilage areas, the map was reconsidered featuring anatomical features and consequently was reduced to 5 regions including the helix (points 1–4), antihelix (points 5–9), concha (points 10–11), tragus (points 13–14) and antitragus (point 12) (Fig. 3b).

Using the more anatomical classification (5-region map) for auricular cartilage, concha cartilage demonstrated a greater Young’s Elastic Modulus than the helix (*p* < 0.01) (Fig. 3b). Specifically the 5 anatomical regions were found to have the following compressive elastic moduli concha 2.08 ± 0.70 MPa, tragus 1.67 ± 0.61 MPa, antitragus 1.79 ± 0.56 MPa, antihelix 1.71 ± 0.63 MPa, and helix 1.41 ± 0.67 MPa. To understand the complex geometry of the auricular structures, a rate of loading irrespective of the thickness of the cartilage was calculated (stress over time). The concha was observed to have a higher rate of loading than the antihelix in compression when accounting for the anatomical structure (*p* < 0.05) (Fig. 3c).

**Figure 4:**
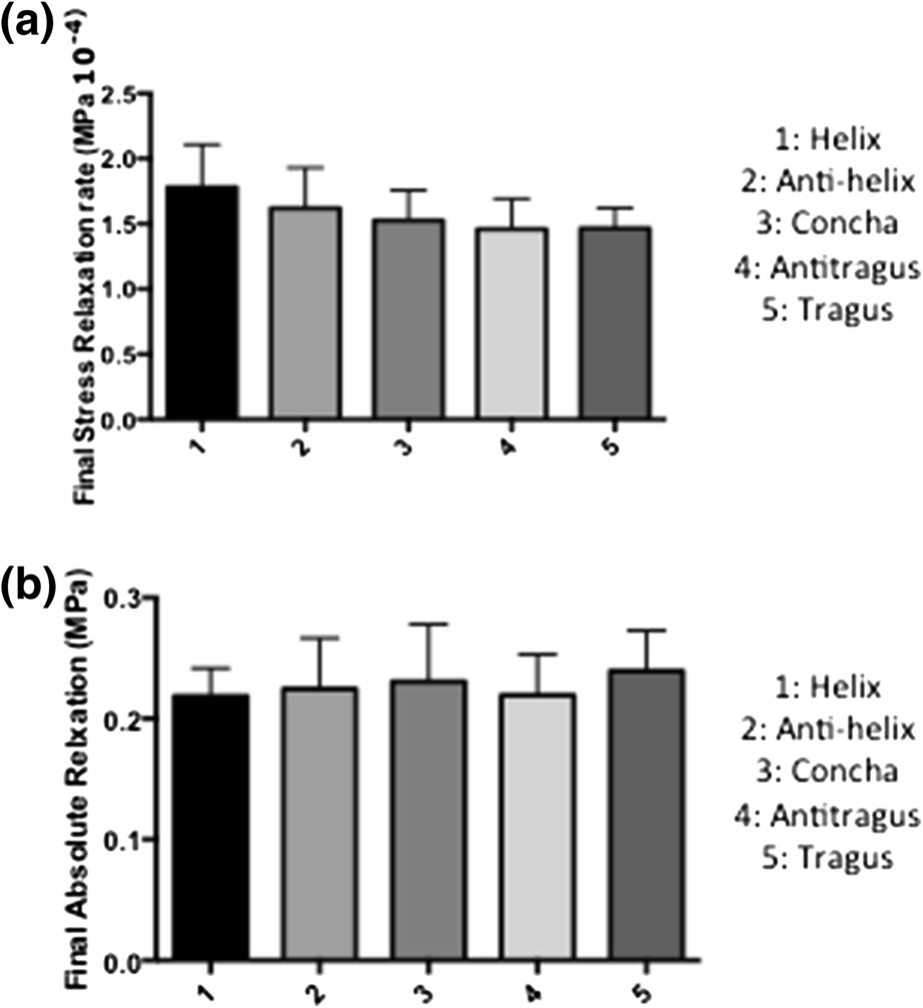
The viscoelastic properties of the auricular cartilage. (a) Stress over the last 200 s of the experiment (rate of relaxation). (b) Final level of relaxation [level of stress at 15 min (end of experiment)].

The final stress relaxation rate was similar for all 5 regions of the auricular cartilage, suggesting that all regions of the auricle had the ability to reach similar load equilibrium over 15 min (helix 1.78 × 10^−4^ ± 0.32 MPa/s, antihelix 1.62 × 10^−4^ ± 0.31 MPa/s, concha MPa/s 1.52 × 10^−4^ ± 0.23 MPa/s, antitragus 1.46 × 10^−4^ ± 0.23 MPa/s and tragus 1.46 × 10^−4^ ± 0.15 MPa/s) Fig. 4a). The final absolute relaxation was also similar between the 5 regions of the auricular cartilage, demonstrating that the auricular cartilages could relax to a similar final stress level (helix 0.21 ± 0.02 MPa, antihelix 0.24 ± 0.04 MPa, concha 0.23 ± 0.04 MPa, antitragus 0.21 ± 0.03 MPa and tragus 0.23 ± 0.03 MPa) (Fig. 4b).

### Histological Testing

**Figure 5:**
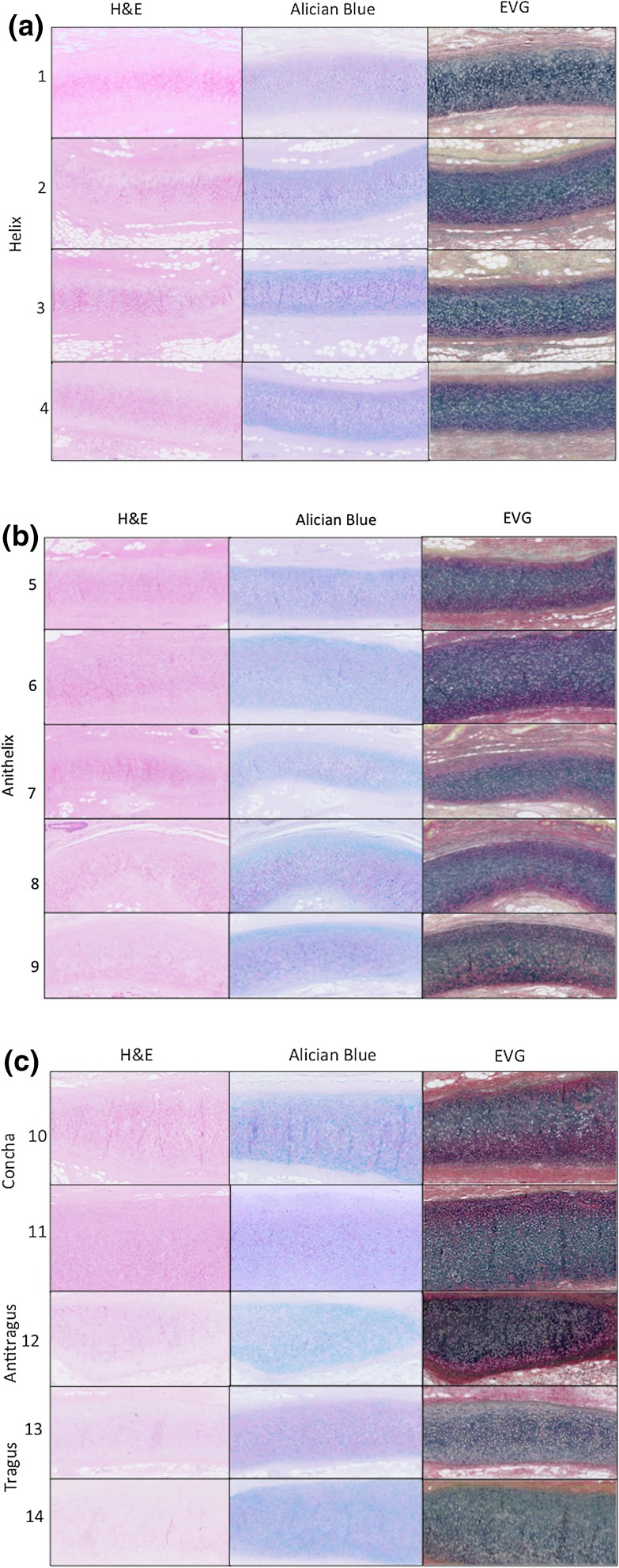
Histological analysis of the auricular cartilages using (a) Haematoxylin and Eosin (H&E) for structure; (b) Alician blue and Periodic Acid-Schiff (PAS) staining for glycosaminoglycan and proteoglycan content; and (c) Elastin Van Gieson (EVG) staining elastin and Collagen type I.

In order to determine the structure of the auricular cartilage components, the tissue was analysed by light microscopy (Figs. 5a–5c). Using H&E, the structure of the auricular cartilages was investigated. All auricular cartilages demonstrated typical elastic cartilage characteristics including chondrocytes immersed within the extracellular matrix (ECM), which was composed of elastic fibres. The EVG stain confirmed elastic cartilage in all cartilage components of the auricle, with positive staining for elastin in the ECM. The chondrocytes showed similar morphology and were evenly distributed throughout the matrix in all auricular cartilage components. ECM content appeared similar between the auricle cartilages with Alician blue staining the acidic polysaccharides including the glycosaminoglycans and PAS staining the proteoglycans.

## Discussion

The aim of this study was to evaluate the compressive mechanical properties of the different anatomical structures of human auricular cartilage. The mechanical properties of the auricular cartilage have not been extensively studied to date. Zahert *et al.* used ten pairs of cartilage specimens from the cavum concha and the tragus from fresh human cadavers.^22^ The young’s modulus was determined by tension calculating a modulus value for concha and tragal cartilage to be 3.4 and 2.8 MPa, respectively, but the difference was not significant. In comparison, in this study the concha was found to demonstrate a greater Young’s Elastic Modulus in compression to the helix. Grellmann *et al.* designed a study to test nasal, costal and auricular cartilage using the following tests: micro-hardness, tension test, compression test, bending test.^4^ However, deeming the auricular cartilage too difficult to test, the authors did not calculate any numerical values for the auricular cartilages.^4^ More recently, Nayyer *et al.* investigated the mechanical properties of the auricle in order to create auricular cartilage matched to human cartilage.^12^ Using six human auricular cartilages, uniaxial tension was applied with a loading speed of 50 mm/min, while tissue thicknesses were measured using an electronic micrometer.^12^ The maximum tensile strength and young’s modulus under tension was calculated with an observed young’s modulus of 5.02 ± 0.04 MPa.^4^ Similarly, Zopf *et al.* evaluated the biomechanical auricular cartilage of four patients, observing that the whole ear exhibited nonlinear strain-stiffening elastic behaviour that is similar to other soft tissues in the body.^24^ To date one study has evaluated the mechanical properties of fresh auricular tissue based on the different region of the auricle. The instantaneous modulus varied according to the region of the auricle, with the helix demonstrating the lowest and the anti-tragus the highest.^13^

It is clear that the main limitation with the literature to date, apart from the minimal studies conducted is the small sample size and lack of regions of the ear that have been tested. We utilised a compressive indentation protocol to test the mechanical properties of human auricular cartilage, having previously used this method to assess human nasal cartilage. Compression was utilised instead of tensile testing as this mimics the physiological forces that the auricle may experience physiologically *in vivo*. Auricular cartilage is compressed during sleeping, physical activity and in response to sounds and is rarely under tensile loads. Furthermore, the main aim of the study was to provide a biomechanical map of the different anatomical elements of the ear and thus tensile testing was not suitable due to limitations in the size of the cartilage required for this mode of testing. Indentation testing is also the most frequently utilised method by which to assess the mechanical properties of cartilage, as the technique does not require special specimen preparation as for confined compression or shear tests and has the added advantage of better resembling the physiological material properties of cartilage.^10^ Furthermore, the non-destructive nature of indentation enabled the ear cartilage in this study to be examined despite its thin and fragile nature.^10^

A particular strength of the mechanical testing protocol used in this study was the detailed 14-area map as there is little mechanical characterisation of auricular cartilage and thus this provides a complete examination of auricular cartilage, providing a benchmark for engineering substitute auricular cartilage. The 5-point anatomical map is also clinically useful when designing anatomical ear implants, as the helix, antihelix, tragus, concha and antitragus are typical landmarks used in auricular reconstruction to provide definition when placed underneath the skin to obtain a satisfactory outcome for the patient.

In this study, the stress–strain data did not present statistically significant different data within the 14 areas, demonstrating the auricle cartilage is a homogeneous structure. The histological mapping showed a similar structure throughout the 14 areas, supporting that the cartilage within the auricle is homogenous. However, the concha cartilage demonstrated a greater Young’s Elastic Modulus than the helix when the auricle was divided into five anatomically structural components, suggesting ultrastructure variances may account for differences in the compressive mechanical properties. In addition to analysing the Young’s Elastic Modulus, the stress-time slope was used to measure the rate of loading of the anatomical ultrastructure (i.e., removal of strain, which normalizes thickness to displacement) (Fig. 3c). The concha had a greater rate of loading than the antihelix when taking into consideration the anatomical structure of the cartilages (*p* < 0.05). One reason for this difference may be that the concha is a curved structure, which can support compressive loads.

The reconstructive surgeon may use synthetic or tissue engineered cartilage to repair auricular defects.^18^ The replacement material is required to provide the anatomical requirements of the auricle including the ability to provide good biocompatibility with the skin. The replacement material must have adequate mechanical properties to prevent deformation of the implant when implanted beneath the skin providing definition of the auricle shape but have similar mechanical properties to the surrounding tissue to prevent stress at the interface and thus implant failure.^8^ Therefore, understanding the biomechanical compressive forces of the native tissue enables replacement materials to be matched to the human native auricular tissue and provide better biocompatibility.^8^,^14^,^21^

Several synthetic materials have been utilised as tissue engineered substitutes for auricular reconstruction including, silicone, Gore-tex and polyethylene. The FDA approved materials polyglycolic acid (PGA) and polylactic acid (PLA) polymers have also been investigated but have shown to induce inflammatory reactions.^15^ Silicone appeared in the 1950s and was the first alloplastic material to gain wide use. It is nonporous, inert and doe not change its shape with time.^1^,^2^,^9^ However after implantation, silicone has shown to form a thick capsule of fibrous tissue leading to poor attachment to the body and high change of movement leading to extrusion of the implant.^1^,^2^,^9^ Polytetraflorethylene or Gore-tex is another material that has been used as a facial implant. It has micropores ranging from 10 to 30 *μ*m leading to limited collagen ingrowth but good biocompatibility and minimal movement.^2^ However, Gore-tex has a high change of deforming with time.^2^ Due to the complications with silicone and Gore-tex, high-density porous polyethylene (HDPP), known as Medpor is the most commonly used synthetic available material for auricular reconstruction. This biocompatible thermoplastic synthetic material has pore sizes of 100–250 *μ*m making it possible for tissue to infiltrate rapidly.^1^ The average Young’s Elastic Modulus of auricular cartilage in this study was found to be 1.66 ± 0.63 MPa in compression. Medpor has been reported to have Young’s Elastic Modulus of 227–307 MPa, far more rigid that auricular cartilage which may contribute to its unnatural feel.^12^ The 100-fold mechanical mismatch between the native tissue and auricular cartilage, can lead to micro-movement between the skin and the implant when subcutaneously implanted, contributing to extrusion. This study provides a reference by which tissue engineered replacements for auricular cartilage should be developed to ensure they have similar biomechanical properties to native tissue and provide better clinical outcomes. It is important to recognise when designing auricular implants, that anatomical structures within the ear can also affect the mechanical properties. It should be considered that the concha cartilage has different mechanical properties when considering the anatomical structure (Fig. 3).

There are limitations in the study design. Despite one of the larger studies to date to examine the mechanical properties of auricular cartilage, the study included male cadavers only due to the availability in tissue specimens. This limitation allowed gender to be removed as a confounding variable, however future studies will analyse the differences in mechanical properties of auricular cartilage due to age and gender. Furthermore, articular cartilage has shown to have nonlinear and anisotropic properties.^6^,^20^ This compression protocol in this study assumed a linear modulus. The protocol tested the same surface of the auricle using one load direction due to limitations in cartilage availability. The ultimate aim of this study was to provide a starting point into understanding the mechanical properties of the human auricle and a linear modulus in one direction achieved this. However, future work will understand the nonlinearity and anisotropy of auricular cartilage using a larger sample size to further complement these initial findings.

Indentation analysis has demonstrated to be useful in characterising the mechanical properties of human auricular cartilage. This study provides a mechanical benchmark by which to manufacture auricular replacements ensuring tissue engineered constructs mimic the biomechanical properties’ of the native tissue.

## Electronic supplementary material

Below is the link to the electronic supplementary material.

Supplementary Figure 1 Loading data of a representative sample. (A) Measuring the initial load resistance data allowed the investigation of the Young’s Elastic Modulus. (B) Measuring the rate of stress relaxation over the last 200 s formulated the final rate of relaxation, and measuring the final stress level at the end of the 15 minutes relaxation period provided the final absolute relaxation (last data point on B) (TIFF 1521 kb)
