# Supplementary figures and images for: Biomechanical Characterisation of the Human Auricular Cartilages; Implications for Tissue Engineering
Source: Ann Biomed Eng. 2016 Jul 14;44(12):3460–7. doi: 10.1007/s10439-016-1688-1 (PMC5112295; doi:10.1007/s10439-016-1688-1)

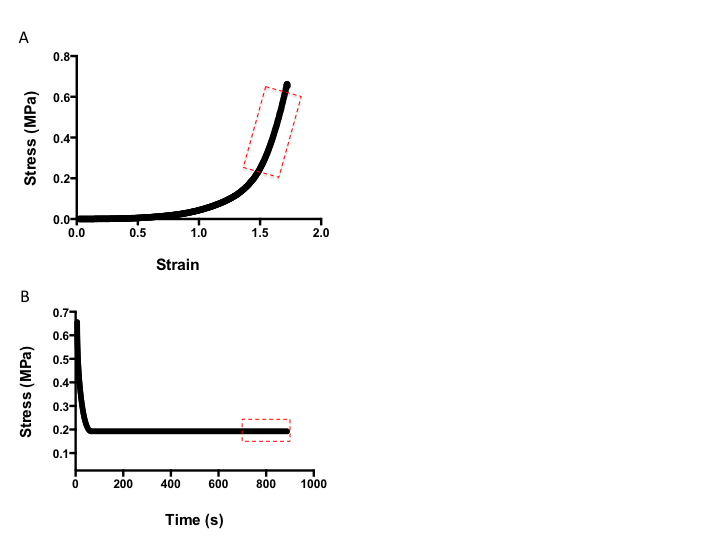

Supplement: Supplementary file 1 — Loading data of a representative sample. (A) Measuring the initial load resistance data allowed the investigation of the Young’s Elastic Modulus. (B) Measuring the rate of stress relaxation over the last 200 s formulated the final rate of relaxation, and measuring the final stress level at the end of the 15 minutes relaxation period provided the final absolute relaxation (last data point on B) (TIFF 1521 kb) [file 10439_2016_1688_MOESM1_ESM.tiff]
